# Basal Septal Hypertrophy

**DOI:** 10.2174/1573403X09666131202125424

**Published:** 2013-11

**Authors:** Mihir A. Kelshiker, Jamil Mayet, Beth Unsworth, Darlington O. Okonko

**Affiliations:** International Center for Circulatory Health, St Mary’s Hospital, NHLI, Imperial College London, UK

**Keywords:** Basal septal hypertrophy, diastolic dysfunction, HFNEF, outflow tract obstruction.

## Abstract

A significant clinical problem is patients presenting with exercise-limiting dyspnoea, sometimes with associated chest pain, in the absence of detectable left ventricular (LV) systolic dysfunction, coronary artery disease, or lung disease. Often the patients are older, female, and have isolated basal septal hypertrophy (BSH), frequently on a background of mild hypertension. The topic of breathlessness in patients with clinical heart failure, but who have a normal ejection fraction (HFNEF) has attracted significant controversy over the past few years. This review aims to analyse the literature on BSH, identify the possible associations between BSH and HFNEF, and consequently explore possible pathophysiological mechanisms whereby clinical symptoms are experienced.

## INTRODUCTION

**Fig. (1):**
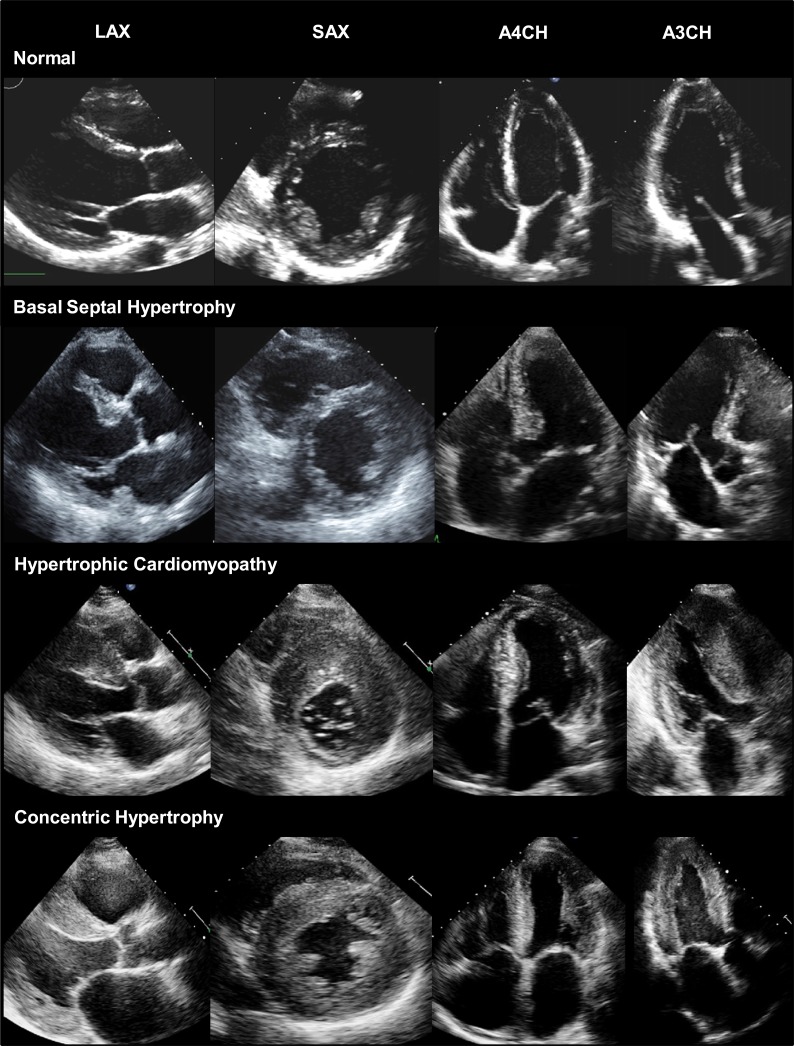
Comparison of normal myocardium, BSH, HCM, and concentric LVH on conventional two-dimensional echocardiography. PLAX = parasternal long axis view, SAX = short axis view, A4CH = apical 4-chamber view, A3CH= apical 3-chamber view.

Focal hypertrophy of the basal inter-ventricular septum is a well-recognized but poorly understood echocardiographic finding (Fig. **1**). In 1978, Maron *et al. *[1] observed that asymmetric enlargement of the inter-ventricular septum could occur independently of hypertrophic cardiomyopathy (HCM), particularly in the presence of systemic hypertension. Despite sample size limitations, the robustness of the authors’ exclusion criteria and cardiac morphometric analyses enabled distinction of the entity of isolated basal septal hypertrophy (BSH) from that associated with other cardiac pathologies in 6% of subjects. Variably termed ‘discrete upper septal hypertrophy’ or ‘sigmoid septum’, BSH subsequently came to be investigated as a substrate for dyspnoea in patients with heart failure and a normal ejection fraction (HFNEF). Here, we review the epidemiology and pathogenesis of BSH and evidence for its pathophysiological effects during ventricular systole and diastole.

## PREVALENCE OF BSH

Prospective studies suggest that up to 10% of cardiovascular cohorts may have isolated BSH. Shapiro *et al. *[2] conducted the first investigation in 1000 patients (age 10-82 years) with diverse cardiovascular diagnoses. After excluding fixed sub-valvular aortic stenosis and HCM, BSH was defined as either a diastolic basal septal thickness 2 standard deviations above the normal mean (greater than 1.4cm), or 50% greater than the thickness of the septum at its mid-point. The authors identified only 8 patients with BSH, but all 8 subjects had evidence of left ventricular hypertrophy (LVH) and aortic valve disease. Thus, no patient with isolated BSH was found. In contrast, Belenkie *et al. *[3] identified isolated BSH (defined as discrepant thickness of the basal septum in at least 2 echocardiographic views) in 7 out of 5582 patients (age 66±11 years). A history of hypertension was evident in 6 of the 7 subjects, whilst it was present in only 2 of the 8 patients in the Shapiro *et al.* study. Using different definitions, later authors reported prevalence rates as high as 10% for isolated BSH [4, 5]. Whilst the variability in prevalence likely reflects the variability in definitions used for BSH, differences in the burden of comorbid hypertension might also have contributed. 

To date, only 3 studies have assessed the prevalence of isolated BSH in otherwise healthy individuals. Diaz *et al. *[6] evaluated 3562 participants (mean age = 58 years) in the Framingham study. BSH was diagnosed on the basis of a visual upper septal ‘knuckle’ with an upper septal thickness >1.4cm in the absence of septal abnormalities (e.g. scarring) that could account for isolated thickening. The authors reported a prevalence of 1.5% with isolated BSH more apparent in elderly individuals with higher systolic blood pressures. Indeed, the prevalence rose to 17.8% in subjects aged 85 years and above. Notably, the authors found that after a mean follow-up of 15-years, the presence of BSH was not associated with an increased risk of cardiovascular disease (hazards ratio = 1.29, 95% CI = 0.77-2.16, P = 0.32) or mortality (hazards ratio = 1.05, 95% CI = 0.71 - 1.55, P = 0.82). Recent studies by Nagaraj *et al. *[7] and Ayoub *et al*. [8] have corroborated these findings. On aggregate, these data accord with the overwhelming clinical impression that BSH is predominately a disease of the elderly and might therefore be a physiological or pathological phenomenon. 

## PATHOGENESIS OF BSH

**Fig. (2):**
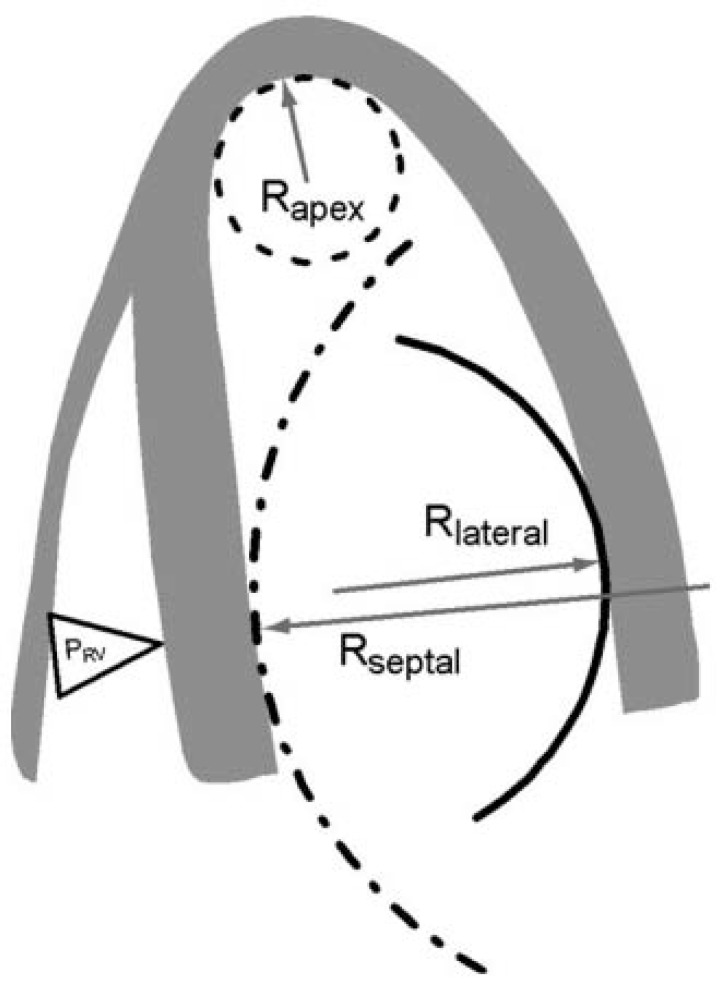
Non-uniformity of the LV (longitudinal) curvature. R_apex_: radius of curvature at the apex; R_lateral_: radius of curvature at the lateral wall; R_septal_ : radius of curvature of the septum; PRV: pressure in the right ventricle. Taken from Baltabaeva *et al.* [13].

The precise mechanisms leading to isolated BSH have yet to be determined but plausible reasons exist as to why the basal septum might be uniquely susceptible to hypertrophy. For example, Laplace’s law states that the larger a vessel’s radius, the larger the wall tension required to withstand internal fluid pressures [9]. Because the longitudinal fibres of the basal septum have some of the largest radii in the human heart, they would be expected to experience the greatest inward component of wall stress [9]. This is compounded by the fact that the basal septum is the last part of the ventricle to be electrically activated, so contractions from other myocardial segments further increase its wall stress (Fig. **2**). Moreover, the additional load created by pressure from the right ventricle exerts additional stress on the septum. Therefore, it is conceivable that the basal septum hypertrophies earlier than other LV regions in response to increased afterload as it already operates under higher loading conditions [1].

Irrespective of whether the basal septum is more susceptible to hypertrophy, there is evidence linking its presence to that of systemic hypertension. Verdecchia *et al. *[10] performed echocardiograms in 496 (age 40-63 years) untreated hypertensive patients and 121 normotensive controls. The majority of patients (61%) had normal LV geometry, 22% had isolated BSH, and 16% had combined septal and posterior LV wall thickening. Duration of hypertension was greater in those with combined thickening compared with those with isolated BSH which, in turn, was longer than those with normal LV geometry. Additionally, 24-hour ambulatory blood pressures were higher in those with isolated BSH than patients with normal LV geometry. Whilst not conclusive, this study suggests that BSH develops earlier in hypertensive patients than other LV structural changes. A similar but smaller study [11] corroborated these findings and demonstrated an increased burden of isolated BSH in hypertensive patients compared with normotensives. Moreover, Sakurai *et al. *[12] demonstrated a significant reduction in BSH after antihypertensive treatment. These findings, coupled with the Framingham study [6] strongly suggest that in the absence of other cardiac abnormalities, it seems both likely and logical that BSH is primarily driven by hypertension. 

Objective evidence for the heightened susceptibility of the basal septum to the structural and functional alterations triggered by hypertension was provided by Baltabaeva *et al. *[13]. Using strain/strain rate imaging, the authors evaluated regional myocardial deformation and longitudinal and radial systolic function in 74 untreated hypertensives (mean age 48.9±1.4years, BP 140-179/90-109mmHg) and 32 age-matched healthy controls. Hypertensive patients had normal radial systolic function and global LV remodelling (left ventricular hypertrophy) with the greatest increase in LV thickness seen at the basal septum. Moreover, significant reductions in myocardial deformation and longitudinal end-systolic and peak systolic stress were seen only at the level of the basal septum, consistent with MRI studies showing impaired septal longitudinal shortening predominantly in the basal region in hypertensives [14]. Additionally, in a recent analysis, Kucukler *et al. *[15] observed that LV hypertrophy developed earliest at the septal base after transverse aortic constriction or exercise in mice. Taken together, these data concord with the notion that BSH is a maladaptive phenomenon, whereby an early physiological response to increased afterload results in local dysfunction. Whether BSH affected global LV systolic and/or diastolic performance was subsequently investigated. 

## POTENTIAL IMPACT OF BSH DURING SYSTOLE 

As an anatomical component of the left ventricular outflow tract (LVOT), a hypertrophied basal septum could alter systolic haemodynamics in a manner akin to the ‘septal bulge’ in HCM that is associated with dynamic LVOT obstruction in the presence of systolic anterior motion (SAM) of the mitral valve [16]. Dynamic LVOT obstruction has also been shown to occur during exercise in the absence of HCM [17], and is now being investigated as a potential cause of ischaemia in diabetes [18], renal failure [19] and tako-tsubo cardiomyopathy [20]. Moreover, it is a potentialsubstrate for dyspnoea in patients with HFNEF. Thus, it is plausible that isolated BSH could precipitate exertional dyspnoea in HFNEF by triggering dynamic LVOT obstruction in the presence of exercise-induced SAM.

Henein *et al. *[21, 22] tested the hypothesis that a bulging basal septum could cause dynamic LVOT obstruction and dyspnoea. The authors conducted dobutamine stress echocardiography (DSE) in 30 patients (mean age 70±12 years) with HFNEF and 15 age-matched controls. At rest, patients had thicker basal septi (2.3 ± 0.5 vs. 1.4 ± 0.2 cm, P< 0.001) and smaller systolic mitral leaflet septal distances (13 ± 4.5 vs. 18 ± 2 mm, P< 0.001) and LV dimensions. At peak stress, LVOT velocities increased from 1.5 ± 0.5 m/s to 4.2 ± 1.2 m/s in patients; mitral leaflet septal distance fell from 13 ±4.5 to 2.2 ± 1.9 mm (P< 0.001) in patients; and SAM appeared in 24 patients (80%) but in no control subjects (P< 0.001). All patients developed limiting dyspnoea approximately 1.5 minutes after peak LVOT velocities and none developed a new wall motion abnormality or mitral regurgitation. Accordingly, the authors concluded that “high velocities, consistent with significant LVOT obstruction, rather than diastolic dysfunction or myocardial ischaemia, corresponded most closely to the appearance of symptoms”. Whilst a clear link between BSH, increased LVOT velocities and dyspnoea during DSE were demonstrated by Henein *et al.*, DSE itself is not physiological exercise. Indeed, Luria *et al. *[23], found that 15 patients who exhibited dynamic LVOT obstruction during DSE failed to reproduce this during exercise echocardiography, calling into question the relevance of DSE findings to the genesis of everyday dyspnoea in HFNEF patients. 

Al-Nasser *et al. *[22] subsequently postulated that if increased LVOT velocities due to BSH were involved in the development of breathlessness, then their deceleration with β-blockade could reduce or negate symptoms. They prospectively studied 15 (age 76±10 years) patients with HFNEF (mean NYHA class 2.7), isolated BSH (>1.6cm), and maximal LVOT velocities >3.5ms^-1^ on DSE. After treatment with atenolol (mean dose 45mg, mean duration 30 months), repeat DSE showed a marked reduction in LVOT velocities (23%, P=0.001) and breathlessness (mean NYHA class fell from 2.8 to 1.5, p<0.0001) in the 11 patients who tolerated beta-blockade Ranasinghe *et al*. [24] substantiated this in a retrospective study. They showed that the use of β-blockers and disopyramide was associated with reductions in mean resting LVOT gradients and NYHA class in dyspnoeic patients with BSH (9 with isolated BSH, 6 with global LV hypertrophy) and resting LVOT obstruction. Taken together, these data strongly link BSH to symptomatic LVOT obstruction. The association of BSH with clinically relevant diastolic dysfunction is less clear.

## POTENTIAL IMPACT OF BSH DURING DIASTOLE 

**Fig. (3):**
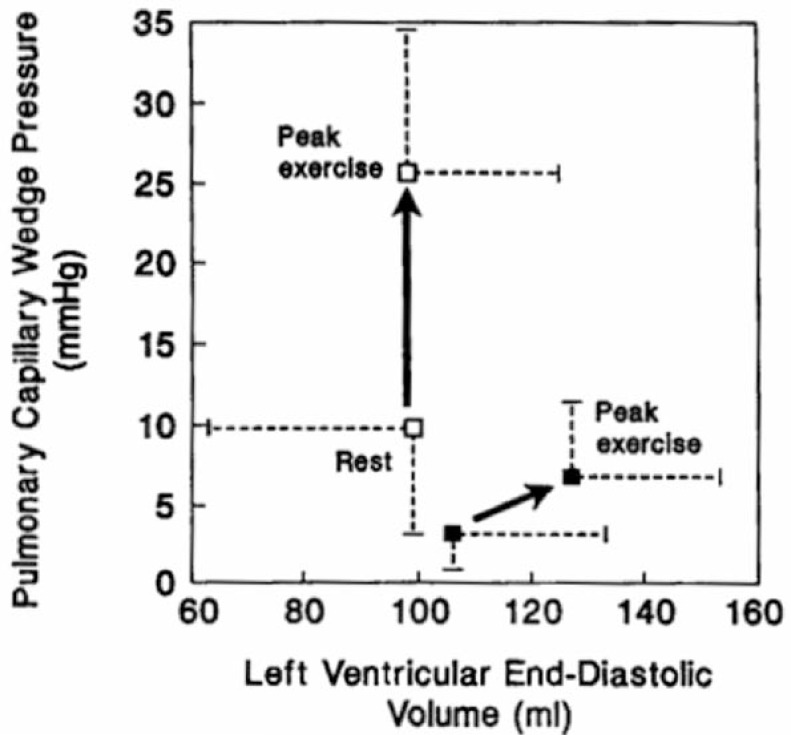
Graph showing association of LVEDV and Pulmonary capillary wedge pressure (LV filling pressure) in normal controls (closed box) and patients with HFNEF (open box). Used with permission from Kitzman *et al.* [25].

Optimal LV diastolic filling is dependent on an active early untwisting process (relaxation) and on the intrinsic passive characteristics (compliance) of the ventricle. Kitzman *et al.* [25] have proposed that abnormalities of LV relaxation and/or compliance can cause a failure of the Frank-Starling mechanism to precipitate symptoms such as dyspnoea and chest pain (Fig. **3**).Whilst it is unlikely that a focal anomaly such as BSH could increase LV passive stiffness in the absence of global hypertrophy, abnormal septal motion due to BSH could hinder energetic myocardial relaxation [26, 27] and trigger symptomatic diastolic impairment. Indeed, in the DSE study by Henein *et al. *[21, 22], diastolic function, as reflected by the E/A ratio, did not differ between controls and subjects at rest but did decrease at peak stress only in patients. This suggests that isolated BSH might be associated with exercise-induced diastolic dysfunction. However, because conventional Doppler echocardiography is heavily load-dependent and cannot differentiate normal and pseudo-normal trans-mitral patterns, other modalities have been utilised to assess diastolic function in BSH. 

Tissue Doppler Imaging (TDI) provides reliable non-invasive measurement of LV relaxation [28] and its indices correlate to invasively determined LV filling pressures [29]. Using TDI, Hung *et al*. [30] found that early diastolic relaxation (E’) in 33 patients (age 50 to 80 years) with BSH (7.1 ± 1.8 cm/s) and 23 patients with aortic stenosis (7.1 ± 1.7 cm/s) were significantly lower compared with those in 25 normal controls (9.3 ± 1.9 cm/s) and higher compared with those in HCM patients (5.4 ± 1.3 cm/s). Additionally, LV filling pressures (E/E’ ratio) were higher in BSH and aortic stenotic patients (10.0 ± 3.1 and 11.3 ± 2.8, respectively) compared with normal controls (7.1 ± 1.6) and lower compared with HCM patients (18.8 ± 7.5). Thus, resting diastolic dysfunction was evident in patients with BSH and, as its magnitude was similar in those with aortic stenosis, BSH was concluded to be a variant hypertrophic response to chronic pressure overload. Again, it is unclear how supposedly global diastolic indices could be affected by a focal aberration such as BSH.

Yalҫin *et al*. attempted to disentangle the relative contributions of systolic (via LVOT obstruction) and diastolic dysfunction to the genesis of dyspnoea during DSE in BSH patients [31, 32]. At rest, patients (n=24) had a narrower LVOT area and poorer diastolic function (lower E/A ratios and prolonged deceleration time) than controls (n=20). As in the study by Henein* et al. *[21], LVOT velocities only significantly accelerated in patients to a mean of 3.3ms^-1^ at peak stress with associated dyspnoea. Unlike Henein *et al.*, the E/A ratio did not change. In a follow-up study using TDI [32], the authors again found isolated changes in indices of systolic function (increase in peak systolic basal septal tissue velocities- 17±3 cm/s vs. 13.7±2.5 cm/s, P< 0.001), and not diastolic function (early/late diastolic tissue velocities), suggesting that abnormalities in systolic performance principally drove dyspnoea in these patients. Whilst this may hold true for all patients with BSH, inconsistencies in our approach to studying BSH and diastolic function restrict the potential to generalise this finding.

## CONTROVERSIES IN BSH RESEARCH AND FUTURE DIRECTIONS

Research on BSH is limited by the variation in criteria used to diagnose it. For example, Henein* et al. *[21] used a mean septal thickness ≥2.3cm whereas Yalҫin *et al. *[31, 32] adopted a thickness ≥1.55cm. Verdecchia* et al. *[10] used a proportional criterion, whereby BSH was present if the ratio of twice the septal thickness divided by the LV internal diameter exceeded 0.45. The implications of these differences are debatable, since in all studies the control groups had significantly thinner septi, and so variation could represent a ‘spectrum’ of hypertrophic change in each individual. However, quantification of the prevalence of BSH is clearly hampered by variations in its definition and reaching a consensus criterion should be a research priority. Whether a threshold level of basal septal thickness exists that best correlates to the development of symptoms in BSH is unknown.

The inconsistency in our approach to diastolic dysfunction particularly hinders proper appraisal of the mechanism(s) via which BSH could trigger symptoms. An editorial by Henein *et al. *[33] outlines the problems effectively. Despite guideline updates to include E/E’ in the diagnosis of diastolic dysfunction and HFNEF, the authors argue that the use of this ratio is still ‘circumventing’ the issue, as it is simply a comparison of two velocities and is therefore only an indirect representation of the dichotomous problem of diastolic dysfunction- a ‘stiff ventricle’ or an abnormally relaxing one . Despite this limitation, E/E’ is of proven clinical utility in varying cardiovascular cohorts [34]. Yet, no study has measured it in patients with BSH and dyspnoea during physiological exercise as opposed to DSE. Future studies that simultaneously measure LVOT velocities and diastolic function using TDI and/or strain imaging during physiological exercise may better illuminate the contribution of these factors to the genesis of everyday dyspnoea in BSH. Such studies should also further appraise potential therapies for symptomatic BSH.

Accumulating evidence suggests that β-blockade is effective in reducing LVOT velocities and breathlessness in patients with BSH during DSE and exercise [22, 24], and in patients with diastolic dysfunction during exercise [35]. However, these studies have either been small; non-randomized; un-blinded;based on older indices of diastolic dysfunction, or conducted in BSH patients with global left ventricular hypertrophy. There is a need for sufficiently rigorous trials assessing the effects of negatively inotropic agents on LVOT velocities, diastolic indices, and symptoms in patients with isolated BSH undergoing physiological exercise.

## SUMMARY

Focal hypertrophy of the basal inter-ventricular septum can be seen in up to 10% of cardiac patients without HCM, being more prevalent in the elderly and hypertensives. Whilst it’s anatomical location plausibly renders it more susceptible to hypertrophy, evidence suggests that the basal septum enlarges mainly due to pressure overload from hypertension. This discrete upper septal hypertrophy is associated with exertional LVOT obstruction and dyspnoea, and appears symptomatically amenable to β-blockade. Whilst diastolic dysfunction likelyalso contributes to symptoms in this condition, the data to date are equivocal. Focused analyses conducted using a consensus definition of BSH, in patients undergoing simultaneous assessment of myocardial systolic and diastolic performance during physiological exercise, are needed to further understand the clinical relevance of this entity.
